# Effect of esketamine nasal drops on pain in children after tonsillectomy using low temperature plasma ablation

**DOI:** 10.3389/fped.2023.1110632

**Published:** 2023-07-17

**Authors:** Qiu Qian, Hua Xian Liu, Yue Qing Li

**Affiliations:** ^1^ Department of Anesthesiology, Suzhou Wujiang District Children’s Hospital, Suzhou, China; ^2^ Department of Anesthesiology, Children’s Hospital of Soochow University, Suzhou, China

**Keywords:** children, esketamine nasal drops, low temperature plasma ablation, tonsillar hypertrophy, tonsillectomy

## Abstract

**Objective:**

To explore the effect of esketamine nasal drops on pain in children after tonsillectomy using low-temperature plasma ablation.

**Methods:**

76 children who underwent tonsillectomy between May 2020 and July 2021, were randomly divided into two groups of 38 cases each. Patients in the control group were treated with conventional medication, while those in the study group were treated with esketamine nasal drops, along with the routine drug treatment. Pain levels of children in the two groups were compared within 1–3 days post-surgery, and the pseudomembrane formation and shedding-off time and recovery time were statistically analyzed.

**Results:**

The pain level of children in the study group was lower than that of the control group 1–3 days post-surgery. The pseudomembrane formation and shedding-off time and recovery time in the study group were shorter than in the control group (*P *< 0.05). There was no significant difference in the incidence of adverse reactions between the two groups, and there were no serious adverse reactions in the two groups (*P *> 0.05).

**Conclusion:**

It is safe to use esketamine nasal drops in children after tonsillectomy using low temperature plasma ablation, and this is found to reduce pain and shorten the recovery time.

## 1. Introduction

Tonsillar hypertrophy is reported as the sensation of a foreign body in the throat, or difficulty when swallowing, and can block the throat leading to obstructed breathing (1, 2). In recent years, low temperature plasma ablation has been widely used in the field of otorhinolaryngology overseas (3). This technique can maintain the safety of local mucosal tissue structures, however, postoperative pain is common. The pain affects sleep, food intake, and daily activities of children, and is not conducive to postoperative recovery (4, 5). Esketamine has strong anesthetic and analgesic effects. However, most of the current studies on esketamine focus on the treatment of depression, and there are few studies on its analgesic effects. Based on this, we explored the effect of esketamine nasal drops on pain in children after tonsillitis using low-temperature plasma ablation, to provide reference for clinical observation. Details of the study are presented in the following sections.

## 2. Data and methods

### 2.1. General profile

With the approval of the Medical Ethics Committee of our hospital, 76 children with tonsillar hypertrophy who were admitted to our hospital from May 2020 to July 2021 were selected as the research participants and randomly divided into two groups of 38 patients each. There were 22 males and 16 females in the control group. Their ages ranged from 3 to 10 years old, with an average age of (6.28 ± 1.13) years; 23 cases had Grade II tonsils and 15 cases had Grade III tonsils. There were 23 males and 15 females in the study group. Their ages ranged from 3 to 11 years old, with an average age of (6.32 ± 1.11) years; 22 cases had Grade II tonsils and 16 cases had Grade III tonsils. The general data of the two groups were similar (*P* > 0.05).

### 2.2. Inclusion and exclusion criteria

(1) Inclusion criteria: ① Patients who underwent low-temperature plasma ablation; ② All children whose families signed the informed consent form voluntarily.
(2) Exclusion criteria: ① Patients with cognitive dysfunction; ② Patients with congenital diseases; ③ Patients with liver and kidney dysfunction; ④ Patients with psychiatric illnesses.

### 2.3. Methods

Tonsillectomy was performed using the US Low Temperature Plasma Surgery System (Smith & Nephew Co., Ltd., UK) and Evac 70° cutter head (using EIC5874-01, Evac 70° cutter head), with an energy level of 7.

Method: 10 min before induction of anesthesia, intravenous injection of esketamine 0.5 mg/kg and dexmedetomidine 1.0 μg/kg were diluted to 10 ml with 0.9% sodium chloride solution. Postoperative diet care was carried out. Half an hour later, the patient was allowed to have cold drinks such as iced milk and ice cream without impurities, which they were advised to swallow slowly to reduce the wound pain and bleeding. Three hours after surgery if there was no bleeding, the patients could eat liquid foods, such as milk, rice congee, soup, and the nursing personnel ensured that the temperature of the food was not too high, and was suitably warm or cold. Some types of fruit and fruit juice containing fruit acid may cause pain as they irritate the wound and this could affect the healing of the wound, hence, patients were advised to consume less of these or not to consume them directly.

#### 2.3.1. Control group

Routine drug treatment—post-surgery, the anti-inflammatory drug cefdinir dispersible tablets (Tianjin Central Pharmaceutical Co., Ltd., GYZZ H20060980) were given orally at 15 mg/kg, three times a day (tid).

#### 2.3.2. Study group

Along with routine drug treatment, patients of the study group were additionally treated with esketamine nasal drops: Esketamine hydrochloride from Jiangsu Hengrui Pharmaceutical Co., Ltd., GYZZ H20193336, 2 drops per nostril at a time, once in the morning and once in the evening daily.

### 2.4. Evaluation indices

We compared the pain severity between the two groups 1–3 days post-surgery using the Visual Analogue Scale/Score (VAS) (6) used for pain assessment. Facial makeup VAS (F-VAS) was based on the VAS with a number of cartoon expressions to make the rating more intuitive and visual, which was suitable for children over 3 years old. There are eleven grades in this scale, with scores from 0 to 10: 0 means no pain, and 10 means the highest pain. A score is selected from 0 to 10 based on how the patient is feeling as an indicator of the level of pain. A score of 1 to ≤3 indicates mild pain that the patient can tolerate. Scores ≥4–6 means that the patient's pain affects sleep but it can be endured. Scores 7–10 indicate severe pain that the patient is unable to bear. All data will be collected by specialized clinical staff. The pseudomembrane formation and shedding-off time and the recovery time were analyzed statistically. The pseudomembrane which has formed is a thin white pseudomembrane with clean surface grows in the wound of the tonsil fossa. The tonsil fossa wound should be evaluated every one hour after surgery by the direct observation of clinical personnel. If the pseudomembrane is cloudy and thickened or accompanied by significant bad breath, the wound may be infected. The criteria for recovery are determined by the subjective feelings of the patient, which means that the patient no longer has the discomfort of the surgical site, can live independently and normally, and can eat normally. The clinical staff followed up the children and their parents every day to inquire about the recovery condition and recorded it.

### 2.5. Statistical methods

SPSS 22.0 software was used to process the data with $\bar{x}\pm s$ as the measurement data; the independent sample *T* test was used to analyze differences between groups and the paired sample *t* test was used for within groups. The data were expressed in percentage, and statistically significant differences were identified using *χ*^2^ test (*P* < 0.05).

## 3. Results

### 3.1. Pain levels of children in both groups 1–3 days post-surgery

The VAS pain scores 1 to 3 days post-surgery in the study group were lower than those in the control group, and the difference was statistically significant (*P* < 0.05) See Table 1.

**Table 1:** Pain levels of children in both groups 1–3 days post-surgery ($\bar{x}$± s*, m*).

Groups	1d	2d	3d
Control group (*n* = 38)	6.74 ± 1.03	5.31 ± 0.95	4.62 ± 0.89
Study group (*n* = 38)	6.25 ± 0.89	4.46 ± 0.73	3.02 ± 0.32
*t*	2.219	4.374	10.429
*P*	0.030	0.000	0.000

### 3.2. Formation time of wound pseudomembrane

The formation time of the wound pseudomembrane was shorter in the study group than that in the control group, and the difference was statistically significant (*P* < 0.05) See Table 2.

**Table 2:** Wound pseudomembrane formation time ($\bar{x}$± s, h).

Control group (*n* = 38)	Study group (*n* = 38)	*t*	*P*
22.73 ± 3.14	18.51 ± 1.96	7.028	0.000

### 3.3. Shedding-off time of pseudomembrane

The shedding off time of pseudomembrane in the study group was shorter than that in the control group, and the difference was statistically significant (*P* < 0.05) See Table 3.

**Table 3:** Pseudomembrane shedding-off time ($\bar{x}$± s, d).

Control group (*n* = 38)	Study group (*n* = 38)	*t*	*P*
10.74 ± 1.92	7.32 ± 1.17	9.377	0.000

### 3.4. Recovery time

The recovery time in the study group was shorter than that in the control group, and the difference was statistically significant (*P* < 0.05) See Table 4.

**Table 4:** Recovery time ($\bar{x}$±s, d).

Control group (*n* = 38)	Study group (*n* = 38)	*t*	*P*
10.89 ± 2.03	7.46 ± 1.22	8.028	0.000

### 3.5. Adverse reactions

One case of dizziness and one case of nausea and vomiting were reported in the control group, while two cases of dizziness and one case of nausea and vomiting were reported in the study group, and the difference was not statistically significant (*P* > 0.05). There were no serious adverse reactions in the two groups.

## 4. Discussion

There are two kinds of tonsillar hypertrophy—physiological hypertrophy and pathological hypertrophy. Most pathological tonsillar hypertrophy, and severe symptoms of physical tonsillar hypertrophy, such as frequent snoring during sleep in children, require surgical treatment. Tonsillectomy has a long history, and currently, the risks associated with surgery are steadily reducing with the continuous improvement of surgical methods and techniques from tonsillectomy to low-temperature plasma ablation. At present, the majority of children's tonsillectomy is performed by using some form of plasma methods as plasma surgery involves less bleeding, shorter operation time, and shorter time of general anesthesia for children, thus the postoperative recovery for children is relatively faster (7, 8).

Low-temperature plasma radiofrequency ablation involves tissue cutting at a relatively low temperature, about 40 °C–70 °C, and can achieve the purpose of hemostasis while cutting, thereby reducing the additional damage to the tissue by the high temperature, relieving the pain of patients and shortening the recovery time (9–15). This technique has been widely used in otorhinolaryngology surgery in China and overseas, and even in orthopedic and arthroscopic surgeries. At present, common otorhinolaryngology surgeries using low-temperature plasma radiofrequency ablation, including palatopharyngoplasty, tonsillectomy, adenoidectomy, epiglottic cyst resection, and vocal cord mass resection, have achieved good therapeutic effects (16–20).

Tonsillectomy is performed in the throat of children—there is intense postoperative pain and there is risk of respiratory tract obstruction. In clinical practice, cefdinir dispersible tablets are generally given orally, which are broad-spectrum antibiotics of cephalosporins and can be generally used for treating inflammation caused by various sensitive bacterial infections. The results of this study have shown that using esketamine nasal drops in children after tonsillitis using low-temperature plasma ablation, can improve pain symptoms and shorten the recovery time. As a novel N-methyl-D-aspartic acid (NMDA) receptor antagonist in the spinal cord, esketamine has a stronger affinity with NMDA receptors and μ opioid receptors clinically, and only half of the dose of ketamine is required to obtain a relatively satisfactory anesthesia effect. In addition, the clearance rate of esketamine is significantly higher than that of ketamine, and the incidence of adverse reactions is reduced. Dexmedetomidine has the effects of sedation and analgesia, anti-anxiety, and inhibition of salivary gland secretion, without significant inhibition on respiration (21, 22). Studies have shown that ketamine combined with dexmedetomidine can provide ideal anesthesia effects for pediatric bronchoscopy and percutaneous kidney puncture biopsy (23–25). Previous studies have confirmed that esketamine has a strong anesthetic effect, which can reduce the amount of analgesic used after surgery, prolong the duration of analgesia, and prevent hyperalgesia caused by opioids. However, the effect of using esketamine nasal drops for pain in children after tonsillitis using low temperature plasma ablation has not been reported yet. There are many methods of clinically administering esketamine. In this study, esketamine was used intranasally. The results showed that the pain in children in the study group was lower than that in the control group 1–3 days post-surgery. The postoperative wound surface pseudomembrane formation and shedding time and the recovery time of the study group were shorter than those of the control group.

In summary, the use of esketamine nasal drops in children after tonsillitis using low-temperature plasma ablation can improve pain symptoms and shorten the recovery time.

## Data Availability

The original contributions presented in the study are included in the article, further inquiries can be directed to the corresponding author.
